# Removal of tetracycline from the aquatic environment using activated carbon: A comparative study of adsorption performance based on the activator agents

**DOI:** 10.1016/j.heliyon.2024.e34637

**Published:** 2024-07-14

**Authors:** Saheed O. Sanni, Oluwayimika Oluokun, Samson O. Akpotu, Agnes Pholosi, Vusumzi E Pakade

**Affiliations:** Biosorption and Water Treatment Research Laboratory, Vaal University of Technology, Private Bag X021, Vanderbijlpark, 1900, South Africa

**Keywords:** Antibiotics, Adsorptive removal, Pine cone, Microwave pyrolysis, Activated carbon

## Abstract

This research focus endeavour to compare the remediation of tetracycline (TC) through activated carbon (AC), crafted utilizing two distinct chemical activators: zinc chloride (ACZ), and potassium hydroxide (ACK), using pine cone biowaste as an effective carbon precursor, followed by microwave-assisted activation. The impact of TC removal by ACZ and ACK adsorbents was thoroughly examined. The influence of pH, adsorbent mass, adsorption isotherms, kinetics, and inclusive thermodynamics were studied. Our results revealed that the interaction between TC and ACZ or ACK adsorbents aligned well with the model of pseudo-second-order kinetics, whilst the Langmuir model fitted the adsorption isotherm data of ACZ and ACK. The ACZ have a maximum adsorption capacity of 327.87 mg/g compared to that of the ACK (283.29 mg/g). Adsorption of TC was facilitated by the suitable pore volume, abundant microporous, and mesoporous structure of these adsorbents. The ACZ adsorbent is abundant in oxygen-containing functional groups, compared to ACK with minimized reactive sites, in bonding with the TC molecules through hydrogen bonding, for faster removal of TC. Our finding from this work further highlights that the synthesized ACZ from pine cones evidenced significant environmental potentials in the elimination of antibiotics from aqueous solution, to promote clean application perspectives.

## Highlights

• Pine Cone was used to prepare porous carbon.
• Two ACs were generated by ZnCl_2_, and KOH chemical activators.
• Activated carbons (ACs) were prepared via a microwave-assisted process.
• The surface area and pore volume of the ACZ have positive effects on TC adsorption capacity.
• Excellent TC adsorption capacity of 327.87 mg/g was obtained by ACZ.

## Graphical abstract

**Figure:**
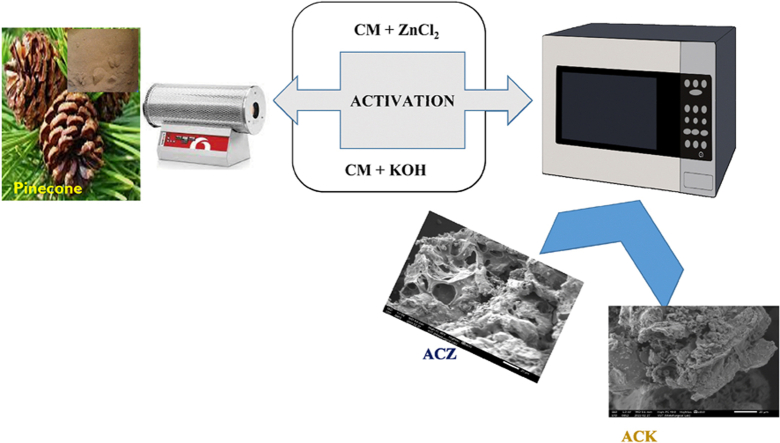

## 1 Introduction

Lately, the hazard and dangers brought about by the mishandling of pharmaceutical antibiotics significantly stand out enough to be noticed in the water resources, worldwide [1]. Irrespective of their low concentration (between ng/L to low mg/L, which is detected in surface, ground, and drinking water) [2], the build-ups of these antibiotics can augment the generation of antibiotic-resistant genes (ARGs), and antibiotic-resistant bacteria (ARB), thus endangering the natural environment, and human health [3,4]. Tetracycline (TC), a common fungicide antibacterial [5], is extensively utilized in medical treatments for humans, and treatments for humans and veterinary medicines to eradicate bacteria and halt the transmission of diseases, owing to its affordability and wide-ranging bactericidal properties [5,6]. Moreover, with the advent of the SARS-CoV-2 (Coronavirus) pandemic around the world, the utilization of TC antibiotics has significantly been explored for controlling the transmission, and treatment of the COVID-19 infection [[7], [8], [9]]. Because of the heightened exploration of TC antibiotics for therapeutic purposes, there is a possible high development and spread of ARGs, thus resulting in mutagenic, and carcinogenic impacts on the human body, in the aftermath of the COVID-19 pandemic [10]. Hence it's critically essential for the advancement of practical economical methods for the removal of the colossal amount of TC generated aftermath of the Covid-19 pandemic.

A few strategies, comprising biodegradation [11], chemical reduction [12], cutting-edge oxidation techniques [13], and adsorption technique [14,15] have been utilized for the removal of TC from aqueous solutions. However, the adsorption process stands out for being a highly effective, simple, low-energy requirements, and low-cost method for removing many pharmaceutical pollutants in contaminated groundwater and wastewater [16,17]. Activated carbon (AC) adsorption as compared to other adsorbents (activated alumina, metal nanomaterials, silicone, and functionalized carbon nanotubes) [[18], [19], [20]] has attracted significant attention for the excellent treatment of TC, and volatile organic compounds (VOCs) [21] owing to their high surface area, specific porous structure, and superior adsorption affinities. However, the commercial ACs adsorbent generated from fossil fuels, are very costly, as such thus paving an alternative pathway for the exploration of abundance, low price, and renewable residual biowaste as ACs precursor in the removal of undesirable organic pollutants. In this regard utilizing biowaste waste residues adds to these materials' sustainability, thus improving the economy [22].

Critically, the performance properties of AC rely upon the preparation method (activation agent, and pyrolysis process) and biowaste precursor. The application of chemical activator agents (i.e. phosphoric acid - H_3_PO_4_, zinc chloride - ZnCl_2_ and alkaline hydroxides (potassium hydroxide – KOH, and sodium hydroxide - NaOH) in ACs preparation is heavily favored in comparison to physical activation, with the lower expense of production, higher quantity of functional groups on the AC surface, along with higher specific surface area, and pore structures from chemical activation strategies [[23], [24], [25]]. Among these activator agents, KOH, and ZnCl_2_ have received broad consideration, due to their positive intercalative influence on pore structure, and specific surface area of generated AC [26]. However, the AC generated from the biowaste residues via these chemical activation process, are typically prepared under the conventional heating in muffle furnace reactors, thus consuming a large amount of energy, activation time, and requirement for large equipment [27]. Additionally, the unevenly heating rate from conventional heating, results in a detrimental effect on the quality, and properties of the prepared AC materials [28]. As such, the exploration of microwave pyrolysis offers potential alternative advantages which involve increased pressure and temperature of the AC material, followed by diffusion of the solvent across the pine cone compared to conventional pyrolysis. The microwave pyrolysis has a uniform, and volumetric heating, lessening the pyrolysis time frame, energy, and gas utilization, which thus enhances the AC overall adsorptive performance [29,30]. According to the literature review, extensive work has been accounted for in the area of microwave-assisted pyrolysis of AC preparation, for adsorptive removal of pollutants [23,31,32].

Yuanyu et al. [33] microwaved rayon fiber felt using (NH_4_)_2_HPO_4_ as activating agent to prepare ACF, for TC adsorptive removal. Using KOH, along with metal oxides as the chemical agent, Martins et al. microwaved pine bark, to prepare AC-metal oxides adsorbent with the adsorption capacity of TC reaching up to 148.24 mg/g [34]. Gurleenjot et al. [35] used a pumpkin seed shells for synthesis of finely grounded – AC adsorbent, with the experiment evidencing that the TC adsorption capacity attained 23.6 mg/g. Jianhua et al. produced magnetic porous biochar from water hyacinth by microwave-assisted hydrothermal, with the adsorption capacity of TC achieving 202.62 mg/g [36]. However, most of this work described above-utilized biowaste residue with lower carbon content, whilst some require higher microwave power in activation of pore structure [33], utilization of multiple chemical activating agents in fine-tuning the physicochemical properties [34,36], and chemical activating agent lost in the filtration process [35], yielding adsorbents with higher time to reach TC equilibrium adsorption capacity. However, the capacity of AC generated from the one-step activation (microwave-pyrolysis) to remove TC from wastewater must be improved. Pine cone (PC) emerges as a bountiful material from nature, readily available and cost-effective, sourced from parks and plantations nestled in the vibrant South African landscape. With its rich carbon composition, lasting strength, harmless nature, and the convenience of being used as it is, without the need for further refinement. PC stands out as a compelling biowaste choice for the production of activated carbon aimed at efficient adsorption treatment [37,38]. Of lately, PC has been employed as a powerful biowaste for the production of activated carbons, and fluorescent carbon dots for catalysis, and sensing applications, for sustainability purposes [[39], [40], [41]]. However, the application of porous carbon derived via a two-step activation process (charring of PC in the furnace, and further microwave pyrolysis of chemically charred PC with KOH/ZnCl_2_) as an adsorbent in the removal of TC antibiotics from wastewater treatment remains unexplored by researchers, therefore the need to undertake this research. Thus, in this work, PC was utilized as our renewable biowaste precursor for synthesizing the activated carbon (ACZ, and ACK) utilizing different activating agents (ZnCl_2_, and KOH), to realize high TC adsorption capacity, within a short equilibrium time. The adsorption properties of the 2 porous carbons were compared via solution pH, adsorbent dosage, adsorption kinetics, and equilibrium aspects of TC adsorptive removal. The adsorption isotherms of TC were also carried out, and well fitted to different isotherm models.

## 2 Experimental procedure

### 2.1 Activated carbon preparation through Zinc Chloride (ACZ), and Potassium Hydroxide (ACK) chemical activation

**Fig. 1:**
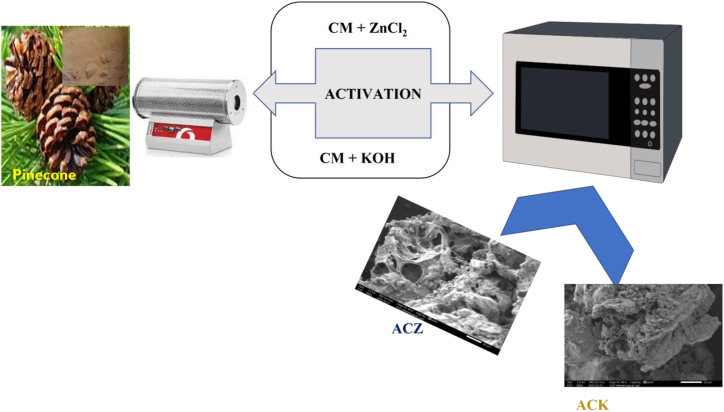
Schematic diagram of the preparation of ACZ, and ACK by microwave pyrolysis method.

In our previously reported procedure [[42], [43], [44]]**,** the activated carbon (ACZ, and ACK) was conducted by implementing a dual-step pyrolysis followed by activation. The ACZ, and ACK adsorbent were prepared through the synthesis process as presented in Fig. 1. Initially, a quantity of 10 g of PC biowaste (collected from Vaal University of Technology, Vanderbijlpark, South Africa) underwent pyrolysis within a tubular furnace (Carbolite Gero) at 600 °C for a duration of 2 h, under a N_2_ flow rate of 50 mL/min at a heating rate of 10 °C/min. After pyrolysis, the furnace was shut down, and cooled down under N_2_ flow till a 200 °C temperature was reached, for carbonized material (CM) collection. Hence, 5 g of CM was combined with 25 mL solution of specific chemical activating agent (2 M ZnCl_2_, and KOH; 99 %, Merck) respectively. The reaction was carried out under ambient temperature, stirred continuously for a duration of 24 h, followed by transfer to an oven at 80 °C for 12 h. The preheated material underwent additional pyrolysis using a microwave power of 400 W for 16 min, under nitrogen atmosphere flow at a flow rate of 50 mL/min [42]. Subsequently, the samples underwent a washing process utilizing 0.1 M HCl (99 %, Sigma Aldrich) and hot distilled water until the filtrate attained a stable pH of 7. The washed samples were subjected to drying at 105 °C overnight, sealed, and preserved in an airtight container for further analysis.

### 2.2 Characterization

The structures, and morphologies of ACZ, and ACK adsorbents were characterized by scanning electron microscopy (Zeiss Leo 1430 VP). A Brunauer-Emmett-Teller analysis (BET, Micromeritics Tristar 3000 analyzer, Australia) utilized for characterizing the surface area, and pore volume of the adsorbents. Additionally, the Fourier transform infrared spectroscopy (PerkinElmer spectrum 400) of ACZ, and ACK adsorbents were performed within the spectral range of 600–4000 cm^−1^. X-ray diffraction (XRD, Bruker diffractometer AXS) was determined using CuKα as the radiation source. The crystal structure was analyzed at 2θ = 10–80°. The point of zero charge (pH-pzc) of the adsorbents was estimated by the pH drift method [42,43,45,46].

### 2.3 Tetracycline (TC) adsorption experiments

#### 2.3.1 Effect of initial pH, mass, and concentration

Adsorption studies was carried out with the two adsorbents developed in Section 2.1. These materials were studied by varying initial parameters such as pH, adsorbent mass, and TC concentration. To investigate the influence of initial pH on TC (98 %, Sigma Aldrich) adsorption, batch experiments on the adsorbents were carried out at different pH values ranging from 3 to 11. The pH of the initial TC solution was adjusted to desired pH using 0.1 M NaOH and HCl solution before the addition of the adsorbents. Next, 10 mg of ACZ, and ACK samples were added sequentially to 50 mL (100 mg/L TC solution) inside a 250 mL polyethylene bottles without light (to minimize self-degradation of the TC antibiotics), and agitated at 220 rpm in an orbital shaker for 2 h. Afterward, the supernatant was filtered using a 0.22 μm polyvinylidene fluoride (PVDF) syringe filter and the TC concentration measured with UV–Vis spectrophotometry (model: T80^+^; PG Instrument; Leicestershire, United Kingdom). at maximum wavelength of 357 nm [45,47]. The effects of adsorbent mass, initial TC concentration, and temperature on the adsorption efficiency of AC adsorbents were studied in the range of 10–150 mg, 25–125 mg/L, and 25–46 °C, respectively. The amount of TC antibiotic adsorbed onto the adsorbents at equilibrium, Qe (mg/g) was calculated according as presented below:

(1) $$Q_{e}=\frac{\left(C_{o-}C_{e}\right)}{m}\;V$$

(2) $$\%\;\mathrm{Removal}=\frac{\left(C_{o-}C_{e}\right)}{C_{O}}\;*100$$

Where Qe is the amount of TC antibiotics uptaken by the prepared activated carbons (mg/g), *C*_*0*_ and *C*_*e*_ (mg/L) represent the initial and final TC concentrations, whilst *m* is the mass of prepared activated carbons (g), and *V* (L) is the volume of TC solution. The adsorption kinetics, isotherm, and thermodynamics models used in this study are presented in Supplementary Information (***SI***).

#### 2.3.2 Desorption and reusability studies

Desorption experiments were conducted on the TC-loaded adsorbents using acidic ethanol as the desorbing agent. For the desorption experiment, 100 mL of acidic ethanol was added to the 1.0 g of TC loaded ACZ and ACK adsorbents, and the suspension was stirred for 2 h at 180 rpm and 25 °C. The solution was filtered and analyzed by UV–Vis spectrophotometry (model: T80^+^; PG Instrument; Leicestershire, United Kingdom), at a maximum wavelength of 357 nm. Four adsorption-desorption experiments were conducted. After every adsorption cycle, the materials were dried at 105 °C for 4 h, then reused for TC adsorption/desorption processes. The TC desorption efficiency was calculated using the following equation:

(3) $$\mathrm{Desorption}\;\mathrm{efficiecy}\;\left(\%\right)=\frac{C_{r}}{{\mathrm{Co}-C}_{e}}\;x\;100$$

Where C_r_ (mg/L) is the TC concentration in the desorption solution and C_e_ and C_e_ (mg/L) are the initial and final TC concentration in the solution before desorption, respectively.

## 3 Results and discussions

### 3.1 Characterization of ACZ, and ACK

**Fig. 2:**
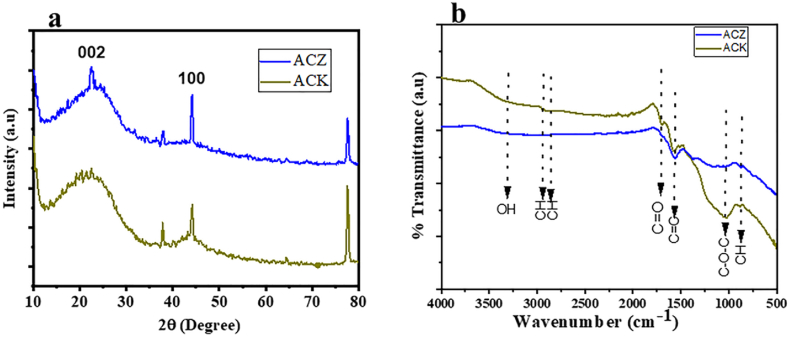
(a) XRD patterns, and (b) FTIR spectra of activated carbon prepared using zinc chloride (ACZ; brown colour), and activated carbon using potassium hydroxide (ACK; blue colour).

XRD was used to analyse the crystallinity, and phase structure of the AC adsorbents as presented in Fig. 2a. The prominent diffraction broad peaks of the ACK, and ACZ samples are observed at 23° and 43° which are considered as confirmations of the carbon material crystal plane (002) and (100), respectively. The presence of sharp (22.1°) and broad peak (23.3°) for ACZ is ascribed to the diffraction peak of aromatic graphitic structure birthed by activation with ZnCl_2;_ while the weak, and broad peak for ACK indicates an amorphous structure with minute graphitization. All the peaks of ACs showed typical XRD patterns, and which are in clear accord with existing research findings [48,49].

The FTIR analysis of the ACZ and ACK materials were conducted to determine the functional groups present in each sample as represented in Fig. 2b. A significant transmittance peak seen at around 3375 cm^−1^ confirms the stretching of the OH functional group on the surface of the prepared activated carbons [5,50] The peak observed at around 1713 cm^−1^ is attributed to the C=O vibrations of the carboxylic group. The C=C functional group stretching ring modes of aromatics is evidenced at around 1576 cm^−1^ [51,52], more pronounced with the ACZ, compared to the ACK carbon material. The peak at 1034 cm^−1^ confirms the existence of C–O–C, groups of the carbonyl, ethers, and alcohol [53] The minute peak in the AC(s) adsorbent around 865 cm^−1^ is showing C–H vibration stretching.

**Fig. 3:**
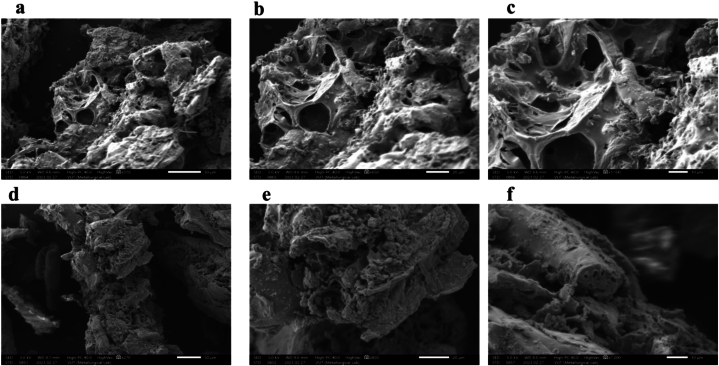
SEM images for (a, b, c) ACZ, and (d, e, f) ACK at different magnifications.

The surface morphologies of ACZ (Fig. 3a, **b, and c**) and ACK (Fig. 3d, e, and **f**) samples were determined using the SEM micrograph on a scale of 50, 20, and 10 μm at different magnifications as presented in Fig. 3a–f. The ACZ showed uniformly open structures with the presence of some rudimentary pores and warped porous structures as compared to the morphology of ACK. The ACK morphology showed slight cracks on the carbons’ surfaces which ultimately led to the generation of porous channel akin to a three-dimensional (3D) hierarchical network, as presented in Fig. 3c and **d**. The significant difference in the enhanced pore development characteristics of ACZ to ACK could be as a result of activation under the microwave pyrolysis condition, and efficient elimination of volatile moisture [54]. Herein, the thermal energy output from the microwave propagates a series of processes comprising.

**Fig. 4:**
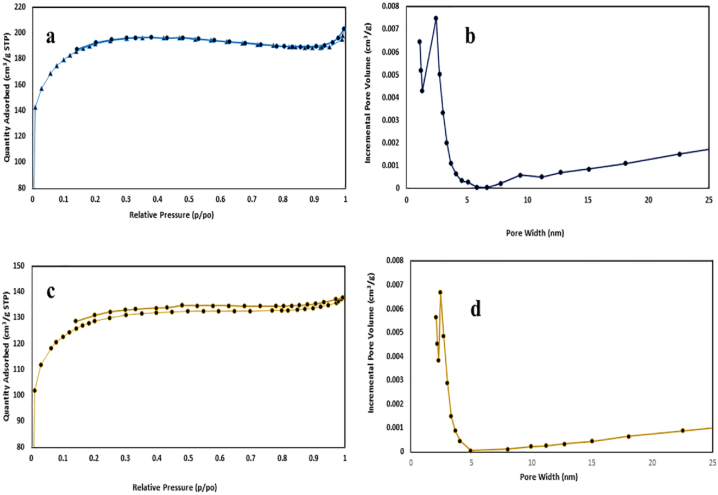
N_2_ adsorption-desorption isotherms, and pore size distributions of (a, b) ACZ, and (c, d) ACK.

The findings of the BET analysis for ACZ and ACK are illustrated in Fig. 4. The ACZ exhibited type I and type II isotherm, indicating the material had a hierarchical pore structure constituted of micropores and mesopores in its structure [26]. On the other hand, the ACK material evident a shape similar to type IIb isotherm [55], which further confirms the ACK has poor microporous, and more mesoporous volume [56]. The total pore volume was 0.116, and 0.101 cm^3^/g, and the surface area were 661, and 438 m^2^/g; respectively for ACZ, and ACK. According to Fig. 4b and **d**, the pore size distribution for ACZ, and ACK are located in the mesopore region (pore size above 2 nm), with an average pore size of 2.43 nm, while the micropore appears approximately between 1.07 and 1.27 nm for ACZ. Overall, the pronounced microporosity, and mesoporosity for the produced ACZ material would significantly create improved structural properties, and several active sites for enhanced adsorptive removal of TC, as compared to ACK.

### 3.2 Influence of initial solution pH, and adsorbent mass

The influence of solution pH on adsorption of TC affects both the surface characters of adsorbents, and the pollutants speciation simultaneously [57]. However, TC functionalities significantly vary with pH changes, as TC exists as H_4_TC ^+^ form when the pH < 3.3, as amphoteric in the form of H_3_TC, at 3.3 < pH < 7.7, while at 7.7 < pH < 9.8, it is anionic as H_2_TC^−^, and at pH > 9.7 it exists in solution as HTC^2−^ [58]. This implies TC charge in solution is dependent on pH.

**Fig. 5:**
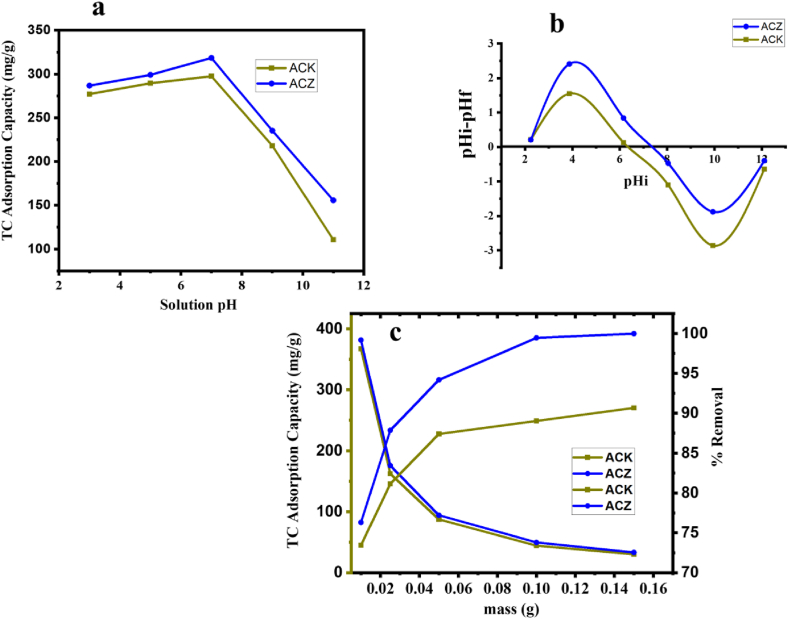
(a) Effect of pH on TC adsorption by ACZ, and ACK (**V: 50 mL, C**_**0**_**: 100 mg/L, adsorbent dosage: 25 mg**). (b) pH-pzc of ACZ, and ACK with various solution pH 2, 4, 6, 8, 10, and 12. and (c) Effect of dosage on TC adsorption by ACZ, and ACK nanocomposite (**V: 50 mL, C**_**0**_**: 100 mg/L,** pH**: 7.0**).

Fig. 5a and b, presented the results of the pH analysis and the corresponding pH-pzc values at which the surfaces of ACZ, and ACK possess a neutral charge of 7.2, and 6.3, respectively. An adsorbent possesses a net positive charge on the surface when the pH of the solution is less than the pH-pzc value, while at pH above the pH-pzc adsorbent surface has an overall negative net charge [[59], [60], [61]]. As illustrated in Fig. 5a, the adsorption capacity of TC with the ACs adsorbent exhibited stable removal, with a gradual increase in sorption capacity from 288 to 316 mg/g for ACZ, and 276–298 mg/g for ACK, as the pH of the solution increases from 2 to 7. Herein, at acidic conditions, both adsorbents and TC species (H_4_TC^+^), in solution are positively charged and thus encounter electrostatic repulsion [62], yielding adsorption capacity being less than the maximum capacity obtained at pH 7 (316 mg/g for ACZ, and 298 mg/g for ACK). Judging that TC speciation is neutral around 3.3 < pH < 7.7, overall, we have an exceptional electrostatic interaction thus enhancing the adsorptive removal of TC [63]. At pH above 7, adsorption of TC decreased and this can be attributed to electrostatic repulsion by the negatively charged adsorbent surface and the TC anions in the solution [34,58]. ACZ, and ACK both adsorb TC in this study, as such the electrostatic interaction between TC and ACZ (highest capacity) is not the sole TC adsorption interaction. Other crucial adsorption interactions comprising pore filling, Π-Π conjugation, and H bonds also influenced the mechanism as presented in Section 3.8.

Fig. 5c shows the influence of ACK and ACZ mass dosage on adsorption of 100 mg/L TC solution at pH 7. The findings indicated that there was a rise in the amount of TC removed from the solution from 32.62 to 87.12 % for ACZ, and 31.24–75.12 % for ACK with an increased in adsorbent mass from 10 to 250 mg. However, the adsorbent adsorption capacity for TC inversely decreased from 315.76 to 35.86 mg/g for ACZ, and 297.79 to 27.68 mg/g for ACK, in connection with the mass of the adsorbent. This observation may be ascribed to an increase in the number of unoccupied adsorption sites present on these adsorbents, along with the presence of a greater number of active surface functional groups [64]. The most effective adsorption dosage was determined to be 30 mg and used in further experiments.

### 3.3 Adsorption kinetic

**Fig. 6:**
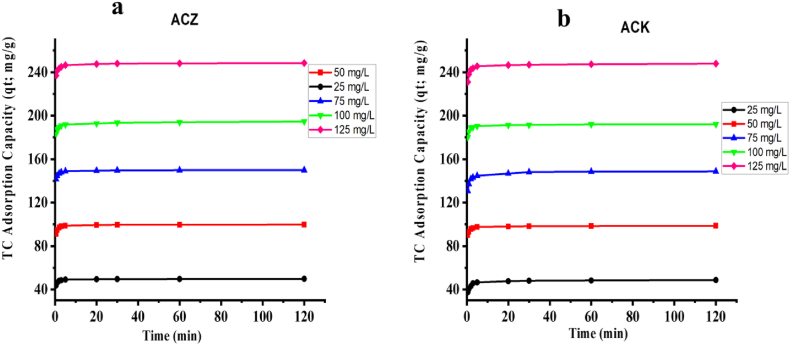
Effect of contact time on TC adsorption capacities with (a) ACZ, and (b) ACK (**V: 50 mL, adsorbent mass: 25 mg,** pH**: 7.0, T: 298 K**).

The adsorption of TC by ACZ and ACK over time at different TC concentrations is presented in Fig. 6a and b. The TC uptake by ACZ and ACK was observed to be rapid at the beginning of the adsorption process, followed by steady increased after 10 min until reaching a nearly constant phase towards the end of the adsorption process. This had been attributed to a large amount of adsorption sites present on the surface of carbon sorbents at the beginning of the adsorption process [58]. The TC uptake was observed to increase with the increase in the TC initial concentration for both adsorbents, which can be ascribed to multilayer adsorption and an increase in concentration gradient driving force otherwise known as diffusion [65]. The TC adsorption capacities of ACZ are slightly higher than that of ACK adsorbent as presented in Fig. 6a and b. This variation in TC removal by ACZ, further justifies the observations highlighted in the SEM, BET, FTIR analysis, and pH studies on the removal process of TC, as compared to ACK. To understand the adsorption process, the adsorbate-adsorbent interaction reactions and mechanisms, non-linear form of four kinetic models (pseudo-first order, pseudo-second order, intraparticle diffusion and elovich kinetic models) were fitted to the experimental data for the adsorption of TC onto ACZ and ACK adsorbent materials. The kinetic fitting results of all the carbon sorbents are presented in Table 1 and 2 The correlation coefficient (R^2^) and the closeness of experimental q_t_ to modelled q_t._ terms are used to conclude on the appropriateness of the fit. The ACZ, and ACK adsorption capacity significantly correlated better with the PSO model, along with the correlation coefficient (R^2^ > 0.9965) and (R^2^ > 9944) in comparison to the PFO model (R^2^＜0.7715) and (R^2^＜0.7504), as presented in Table 1 and 2. Furthermore, the theoretical adsorption capacity of the fitting equation was basically in good agreement with the experimental equilibrium adsorption data for the pseudo second order model than the pseudo first order model. The fitting of the data to pseudo second order model suggest that the adsorption process was controlled by exchange of surface electrons between the adsorption sites of the adsorbents and the TC molecules in solutions resulting in chemisorption mechanism [66]. The rate constants, *k*_*1*_ and *k*_*2*_, were observed to be higher for ACZ than for ACK adsorbent suggesting a more rapid kinetic uptake for ACZ than for ACK adsorbent. The results suggested that the abundance of active sites on the surface of ACZ adsorbent significantly played a major role in the reaction rate, whilst chemical adsorption was the rate-limiting step [67]. In addition, the pore-filling based on physical interaction also significantly contributes to enhanced TC adsorption by ACZ adsorbent.

**Table 1:** Kinetics datas estimated for the adsorption of Tetracycline onto ACZ.

Kinetic model	25 mg/L	50 mg/L	75 mg/L	100 mg/L	125 mg/L
**ACZ**					
**Pseudo-first order**					
*Model q*_*t*_ (mg/g)	49.0010	98.5846	148.5571	191.9060	246.2001
*k*_*1*_ (min^−1^)	4.2868	5.0083	6.0285	6.115	6.4890
*r*^*2*^	0.7715	0.7593	0.6837	0.6407	0.6489
**Pseudo-second order**					
*Exp. q*_*t*_ (mg/g)	49.9	99.8	149.99	194.6	248.5
*Model q*_*t*_ (mg/g)	49.7740	99.7262	149.7139	193.3923	247.7896
*k*_*2*_ (g/mg min)	0.2845	0.2054	0.2208	0.1747	0.1655
*r*^*2*^	0.9949	0.9965	0.9853	0.9561	0.9671
**Elovich**					
α (g/mg.min)	1.2898 E 22	5.3469 E 31	1.2613 E 43	3.4747 E 43	6.1449 E 43
β (g/mg)	1.09766	0.7661	0.6828	0.5325	0.4159
r^2^	0.7152	0.7113	0.7485	0.8487	0.7357
**Intraparticle Diffusion**					
*K*_*ip*_ (mg/g min^0.5^)	0.3843	0.5471	0.5571	0.8028	0.8045
*C (mg/g)*	46.7640	95.4454	145.4959	187.6705	241.8988
*r*^*2*^	0.4363	0.4285	0.4762	0.5995	0.5362

**Table 2:** Kinetics datas estimated for the adsorption of Tetracycline onto ACK.

	25 mg/L	50 mg/L	75 mg/L	100 mg/L	125 mg/L
**ACK**					
**Pseudo-first order**					
*Model q*_*t*_ (mg/g)	46.6819	97.2318	145.3217	190.2427	245.0334
*k*_*1*_ (min^−1^)	2.8693	5.0702	4.4086	5.7895	5.6092
*r*^*2*^	0.6886	0.6957	0.6853	0.7504	0.7348
**Pseudo-second order**					
*Exp. q*_*t*_ (mg/g)	48.8	98.69	148.8	192.1	247.9
*Model q*_*t*_ (mg/g)	48.1590	98.3964	147.7048	191.7860	247.2387
*k*_*2*_ (g/mg min)	0.1276	0.2075	0.0970	0.1591	0.1113
*r*^*2*^	0.9612	0.9854	0.9720	0.9944	0.9935
Elovich					
α (g/mg.min)	5.1852 E 9	3.5518 E 30	1.0813 E 21	1.153 E 43	2.1771 E 41
β (g/mg)	0.5253	0.7481	0.344	0.5318	0.3956
r^2^	0.9281	0.7559	0.8303	0.7234	0.7457
**Intraparticle Diffusion**					
*K*_*ip*_ (mg/g min^0.5^)	0.8410	0.5718	1.2867	0.7480	1.0823
*C (mg/g)*	41.8214	94.0392	138.2405	186.0397	238.9898
*r*^*2*^	0.5543	0.4744	0.5590	0.4528	0.4699

### 3.4 Adsorption isotherm

The adsorption isotherm activities of TC unto the ACZ, and ACK were further analyzed, whilst the model fittings (Langmuir and Freundlich) were applied to study the interaction between the TC antibiotics, and the adsorbents, as presented in Fig. S1. The results show that the experimental findings demonstrated a strong alignment with the Langmuir model for TC adsorption onto ACZ and ACK adsorbents, with high (R^2^＞0.9962) for ACZ and (R^2^＞0.9988) for ACK, compared to the Freundlich model with (R^2^＜0.9932) for ACZ and (R^2^＜0.9965) for ACK adsorbent, as presented in Table 3. Higher R^2^ values for Langmuir model for both adsorbents, shows that the Langmuir isotherm better fitted the experimental data than Freundlich isotherm, suggesting that the adsorption sites on both adsorbents are homogenous, forming a monolayer coverage of adsorbed TC on the surface. The values of monolayer capacity were observed to decrease as the adsorption temperature increased from 298 to 314 K for both adsorbents and ACZ adsorbent had higher values as compared to ACK adsorbent. The values of K_L_, on the other hand, was observed to increase with an increase in temperature for both adsorbents. This shows that the adsorption of TC by both adsorbents is not favored at a higher temperature. Also, the Kf values (a measure of the degree of adsorption) in the Freundlich model, decrease with an increase in temperature for both adsorbents. As more adsorption occurs at lower temperatures with higher Kf values as presented in Table 3 (see Table 4).

**Table 3:** Equilibrium data estimated for the adsorption of Tetracycline onto ACZ and ACK.

Isotherm model	298 K	304 K	309 K	314 K	319 K
**ACZ**					
**Langmuir**					
*Qmax* (mg/g)	327.8689	318.4713	312.50	285.7143	232.5581
*K*_*L*_ (L/mg)	1.95 × 10^−4^	2.93 × 10^−4^	3.81 × 10^−4^	5.52 × 10^−4^	7.21 × 10^−4^
*r*^*2*^	0.9962	0.9893	0.9897	0.9960	0.9982
**Freundlich**					
*KF (mg/g) (mg/L)1/n*	0.1228	0.0720	0.0375	0.0033	0.011
*n*_*F*_	0.6687	0.6999	0.7136	0.7292	0.7459
*r*^*2*^	0.9932	0.9823	0.9844	0.9920	0.9938
**ACK**					
**Langmuir**					
*Qmax* (mg/g)	283.2861	280.8989	275.4821	264.5503	259.7403
*K*_*L*_ (L/mg)	8.33 × 10-5	1.16 × 10-4	1.41 × 10-4	1.68 × 10-4	2.19 × 10-4
*r*^*2*^	0.9978	0.9903	0.9988	0.9985	0.9841
**Freundlich**					
*KF (mg/g) (mg/L)1/n*	0.2185	0.1915	0.1782	0.1659	0.1384
*n*_*F*_	0.5783	0.5517	0.5804	0.5789	0.6078
*r*^*2*^	0.9865	0.9509	0.9754	0.9740	0.9547

**Table 4:** Adsorption thermodynamics of tetracycline onto ACZ, and ACK.

Adsorbent	Temperature (K)	Gibb's free energy *ΔG*° (kJ/mol)	H (kJ/mol)	S (JK^−1^mol^−1^)
ACZ	298	−3.48		
	304	−2.78		
	309	−2.25	−40.52	−123.97
	314	−1.54		
	319	−1.00		
ACK	298	−5.06		
	304	−4.37		
	309	−2.25	−24.13	−64.41
	314	−3.93		
	319	−3.69		

Furthermore, the maximum adsorption capacity (Q_max_) of TC for the ACs adsorbent was 327.87, and 283.29 mg/g, for ACZ, and ACK. The enhanced adsorption properties of ACZ onto TC antibiotics are credited to their unique morphology. Also, the ACZ has more pore volume, and abundant microporous, and mesoporous structure, thus making it favorable for TC molecules to get into the porous of ACZ. According to this analysis, the prepared ACZ is a promising adsorbent material that can be employed to treat pharmaceutical-contaminated water.

### 3.5 Thermodynamic parameters

The adsorption thermodynamics of TC onto ACZ, and ACK adsorbents are shown in Table 3. The negative values of ΔG° varied from −3.48 to −1.00 kJ/mol and −5.06 to −3.69 kJ/mol for ACZ and ACK respectively. This data here indicated that the adsorption of tetracycline occurred spontaneously, with a rise in temperature resulting in more negative ΔG° values [35,68]. This confirms that the adsorption became increasingly favorable for both ACs adsorbent materials as temperature increases from 298 to 319 K. The values of enthalpy and entropy of the system were extrapolated from the plot of lnK against the inverse of temperature (I/T). The negative ΔH°, and ΔS° values suggest that the adsorption process is exothermic and leads to a reduction in randomness during adsorption [69]. Given the exothermic nature of the adsorption process and the negativity of ΔG°, it can be concluded that enthalpy governs the adsorption process.

### 3.6 Comparison of adsorption capacities with similar materials

Table S1 presented the adsorption capacity of TC onto ACZ, and ACK, in comparison with other carbon materials generated from different biowaste in the removal of TC. The removal adsorption capacity of TC by ACZ, and ACK presented maximum sorption capacities (*Q*_*max*_) values of 327.87 mg/g, and 283.29 mg/g, which was particularly high, and acceptable. Overall, these adsorbents can be efficiently applied in the environmental remediation of an aqueous solution containing pharmaceuticals.

### 3.7 Adsorption mechanism Insight onto TC removal by carbon materials

**Fig. 7:**
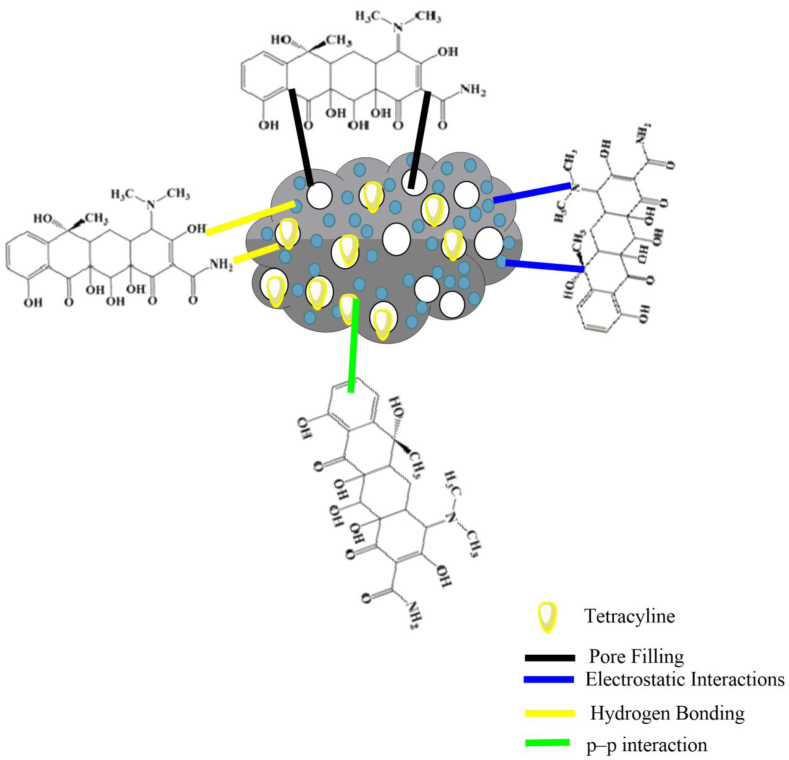
Illustration of TC adsorption interaction mechanism onto ACZ sample.

The adsorption interaction mechanism involved with TC onto the ACZ material was significantly influenced by pore filling effects, hydrogen bonding, π–π electron donor–acceptor (EDA) interactions, and electrostatic interactions., respectively as presented in Fig. 7. Considering, the SEM image observation of ACZ material in Fig. 3a–b with well-developed pore structure, and high pore diameter from BET results, would thus significantly contributes more to TC adsorption onto the AC materials. Herein, the pore-filling attributes were enhanced due to the smaller size dimensions of the TC molecule (1.26 × 0.69 × 0.76 nm), by accessing the narrow, and wider pore structure of the ACK and ACZ adsorbents with pore sizes more than 2 nm, thus ensure the rapid transfer of adsorbate onto the carbon materials [70]. As indicated by the FTIR analysis, the AC materials possessed numerous functional groups on their surfaces, as such particular interactions comprising hydrogen bonds, and π–π EDA interactions tend to occur.

Based on the FTIR findings of ACZ, and ACK (Fig. S2), the O–H stretch peak (most especially with the ACZ adsorbent) at 3378 cm^−^^1^ becomes broader after the adsorption process with TC. Also, the appearance of a –CH group from the alkyl at 2920 and 2812 cm^− 1^ was also visible with ACZ after adsorption interaction with the TC molecule as presented in Fig. S2. These functional groups in ACZ, and ACK would interact via hydrogen bonding with the amino groups present on the TC molecule [69]. However, the C–O–C group at 1030 cm^− 1^ for ACZ was more broaden, and sharper compared to ACK, thus confirming their reaction with the phenolic groups on TC, can be attributed to hydrogen bonding. The weak –C=O stretching vibration peak (for ACZ) shifted to a lower frequency after adsorption, indicating that π–π EDA interactions occurred [71,72].

### 3.8 desorption and reusability studies

**Fig. 8:**
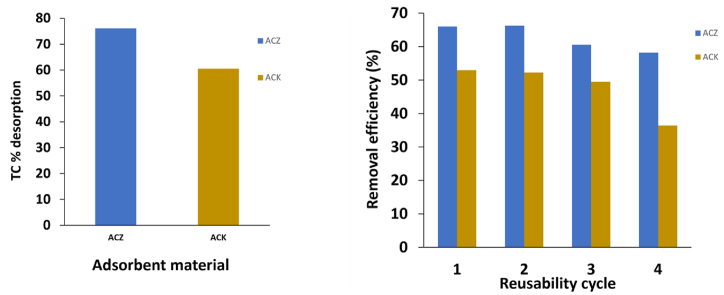
(a) Desorption profile and (b) Regeneration of TC onto ACZ and ACK after 4 cycles of adsorption-desorption.

Desorption and reusability studies of an adsorbent during the adsorption-desorption cycles are very important because it can greatly reduce the material cost which is significant for industrial applications. In this regard, acidic ethanol was used as the desorbing agent and TC adsorption-desorption cycles on ACZ and ACK was repeated 4 times, and the results are shown in Fig. 8a and b. It is observed that acidic ethanol was effective in the desorption of TC with desorption efficiency of 76.02 % and 60.48 % for ACZ and ACK, respectively. The TC percentage adsorption onto ACZ slightly reduced with increasing regeneration cycles up to 4 cycles while the TC percentage adsorption onto ACK slightly reduced with an increase in regeneration cycles up to 3 cycles then sharply reduced as regeneration cycles increase to the 4^th^ cycle. The reduction in TC percentage removal with an increase in regeneration cycles is due to the decrease in active sites of the adsorbent as it is applied several times and the structural damage of surface functional groups on the adsorbent [73]. The regeneration results, however, show that both adsorbents were effectively regenerated and reused for the uptake of TC pollutant from an aqueous solution. ACZ adsorbent can be reused for at least four adsorption-desorption cycles, while ACK adsorbent can be reused for at least three adsorption-desorption cycles without significant loss of its percentage removal. This would effectively reduce the total cost of the adsorbent.

## 4 Conclusions

Herein, activated carbons prepared from low-cost agricultural pine cone biowaste, in a solution containing ZnCl_2_ and KOH as chemical activators, subsequent activation was carried out with the assistance of microwave pyrolysis, were assessed for TC adsorptive removal. The prepared ACZ has a rich pore structure, high specific surface area, and abundant oxygen-containing functional groups on the surface as reactive sites for adsorption, as compared to ACK adsorbent. The prepared ACZ showed to be effective (327.87 mg/g) compared to the ACK (283.29 mg/g) for the elimination of TC antibiotic in an aqueous solution. The PSO kinetic model, and the Langmuir isotherm model significantly well-represented TC adsorption capacity onto the prepared ACZ, and ACK. The adsorption mechanisms of TC onto the prepared ACZ were significantly influenced by the pore filling effects, hydrogen bonding, π–π electron donor–acceptor interactions, and electrostatic interactions. Overall, the ACZ prepared from pinecone biowaste is an accessible and inexpensive adsorbent material with crucial role in mitigating environmental pollution.

## Funding

This research was supported by the 10.13039/501100013222Vaal University of Technology Research Directorate.

## Ethics approval

Not Applicable.

## Consent to participate

All authors consent to participate.

## Consent for publication

All authors have consented to publication.

## CRediT authorship contribution statement

**Saheed O. Sanni:** Writing – review & editing, Writing – original draft, Methodology, Data curation, Conceptualization. **Oluwayimika Oluokun:** Writing – review & editing, Writing – original draft, Resources. **Samson O. Akpotu:** Writing – review & editing, Visualization, Validation, Formal analysis, Data curation. **Agnes Pholosi:** Writing – review & editing, Writing – original draft, Methodology, Data curation. **Vusumzi E Pakade:** Writing – review & editing, Writing – original draft, Methodology, Investigation, Conceptualization.

## Declaration of competing interest

The authors declare the following financial interests/personal relationships which may be considered as potential competing interests: Saheed reports administrative support and equipment, drugs, or supplies were provided by 10.13039/501100013222Vaal University of Technology Faculty of Applied and Computer Sciences. None reports a relationship with none that includes:. None has patent none pending to none. The authors declare that they have no known competing financial interests or personal relationships that could have appeared to influence the work reported in this paper. If there are other authors, they declare that they have no known competing financial interests or personal relationships that could have appeared to influence the work reported in this paper.
