# Supplementary material for: Removal of tetracycline from the aquatic environment using activated carbon: A comparative study of adsorption performance based on the activator agents
Source: Heliyon. 2024 Jul 14;10(14):e34637. doi: 10.1016/j.heliyon.2024.e34637 (PMC11315132; doi:10.1016/j.heliyon.2024.e34637)
Supplement: Multimedia component 1 [file mmc1.docx]

**Supplementary Material**

**Removal of tetracycline from the aquatic environment using activated carbon: A comparative study of adsorption performance based on the activator agents**

**Saheed O Sanni^*^, Oluwayimika Oluokun, Samson O Akpotu, Agnes Pholosi,Vusumzi E Pakade**

Biosorption and Water Treatment Research Laboratory, Vaal University of Technology, Private Bag X021, Vanderbijlpark 1900, South Africa

* Corresponding author: [mosqit.saheed@gmail.com](mailto:mosqit.saheed@gmail.com); [vusumzip@vut.ac.za](mailto:vusumzip@vut.ac.za)

**S 1.0. Kinetic, isotherm, and thermodynamic**

The pseudo-first-order and pseudo-second-order models [1, 2] were used to fit the kinetic data. The non-linear kinetic models were modeled against the experimental data using kyplot software. The mathematical equations of these respective models employed in this study are presented below in Equations 1, and 2.

| $\text{q}_{\text{t}}\text{=}\text{q}_{\text{e}}\text{.}\text{[}\text{1-exp}\text{(-}\text{k}_{\text{1}}\text{t}\text{)]}$ | (1) |
| --- | --- |
| $q_{t}=\frac{k_{2}.q_{e}^{2}.t}{1+q_{e}.k_{2}.t}$ | (2) |

Herein, *q_t_*, *q_e_* are the amount of TC adsorbed by ACZ, or ACK at reaction time *t* and the equilibrium respectively (mg/g); *t* is the contact time (min); *k_1,_ and k_2_* are the pseudo-first-order rate constant (min^-1^), and pseudo-second-order rate constant (g / mg. min) respectively.

Also, the non-linear form of elovich, and intraparticle diffusion are presented below:

$q_{t}=\frac{1}{\beta}In(1+\alpha\beta t)$ (3)

$q_{t}=k_{ip}t^{0.5}+C$ (4)

*Where α* (g /mg min) and *β* (g/mg) are the initial adsorption rate and the Elovich constant, respectively. K_ip_ are intra-particle diffusion models (g/mg min), respectively, and C (mg/g) is the constant.

1.1 **Isotherm model analysis**

The Langmuir, and Freundlich, non-linear isotherm models [3-5] were employed to analyze equilibrium data, as presented in equations 3, and 4, respectively. These were modelled with the experimental data using origin software.

| $\frac{C_{e}}{q_{e}}= \frac{1}{bq_{m}}+\frac{C_{e}}{q_{m}}$ | (3) |
| --- | --- |
| $\ln q_{e}=ln K_{F}+ \left( \frac{1}{n} \right)\ln C_{e}$ | (4) |

Where *q_e_* is the TC amount adsorbed at equilibrium (mg/g); *C_e_* is the TC concentration at equilibrium (mg/L); *Q_max_* is the maximum sorption capacity of the adsorbent (mg/g); K_L_ is the Langmuir equilibrium constant (L/mg); K_F_ is the Freundlich equilibrium constant (mg/g)(mg/L); n_F_ is the exponent of Freundlich model, respectively, (n_F_ is dimensionless).

The TC adsorption performance by ACZ, and ACK was analyzed between temperatures ranging from 298 to 319 K. The thermodynamic parameters comprising Gibb’s free energy change (*∆G°*, kJ/mol), enthalpy change (*∆H°*, kJ/mol), and entropy change (*∆S°*, J/mol K), were evaluated separately with these equations below:

$lnK_{c}=\frac{-\Delta H}{RT}+\frac{\Delta S}{R}$ (7)

$\Delta G=-RTlnK_{c}$ (8)

$K_{c}=\frac{q_{e}}{C_{e}}$ (9)

Where *R* is the universal gas constant (8.314 J K^-1^ mol^-1^); *T* is the absolute temperature (Kelvin); K_c_ is the thermodynamic equilibrium constant, which was calculated according to equation 7, K_e_ is dimensionless.

**Table S1** Comparison of adsorption capacities of different adsorbents for tetracycline removal

| **Adsorbents** | **pH** | **Temperature (K)** | **Co (mg L^−1^)** | **Q_m_ (mg g^−1^)** | **Equilibrium time**  **(min)** | **References** |
| --- | --- | --- | --- | --- | --- | --- |
| Activated Carbon from Cotton Cloths (ACCs) | 3 -9 | 297 | 5 – 100 | 109.12 | 180 | [6] |
| Modified Biogas Residue Biochar (CABRC) | 3 - 11 | 298 - 318 | 5 – 100 | 94.2 | 1400 | [7] |
| Tyre Pyrolysis Char (TPC) | 6.5–7.5 | 298 | 50 – 300 | 312 | 3000 | [8] |
| Human Hair-Carbon (HHC) | 3–13 | 30 - 50 | 25–355 | 210.18 | 1500 | [9] |
| Fish Scales Carbon (FSC) | 3–13 | 30 - 50 | 20 – 100 | 108.02 | 1500 | [10] |
| Electrolytic Carbon (EC) | 2 - 9 | 25 - 35 | 30 – 250 | 393.5 | 260 | [11] |
| NaOH-Activated Macroalgae Carbon (ENAC) | 2 - 11 | 30 - 50 | 50 – 500 | 381.584 | 900 | [12] |
| Peanut Shell Prepared by Sulfuric Acid Reflux (PNS-SO_3_H) | 3 – 8.5 | 297 | 50 – 400 | 303 | 360 | [13] |
| Saw Dust Prepared by Sulfuric Acid Reflux (SD-SO_3_H) | 3 - 9 | 25 - 65 | 100 – 400 | 270 | 360 | [14] |
| Activated Carbon From Tomato (TAC) | 5.7 | 298 - 308 | 200–800 | 500 | 480 | [15] |
| Activated Carbon- Derived From Sugar Cane Bagasse (ZBAC) | 2 - 10 | 298 - 308 | 240 -480 | 239.6 | 250 | [16] |
| Biochar Derived From Rice Straw (RSBC-700) | 2.5–10.5 | 308 | 20 – 50 | 50.74 | 1400 | [17] |
| KOH-Activated Biochar Derived From Reed Plants (RBC) | 3 - 9 | 15 - 40 | 10 – 45 | 173.61 | 1400 | [18] |
| Activated carbon from Potassium Hydroxide (ACK) | 3 - 11 | 298 – 319 | 25 – 125 | 327.87 | 120 | Present Work |
| Activated carbon from Zinc Chloride (ACZ) | 3 - 11 | 298 – 319 | 25 – 125 | 283.29 | 120 | Present work |

**Fig. S1.** Adsorption equilibrium isotherm of TC onto (a) ACZ, and (b) ACK


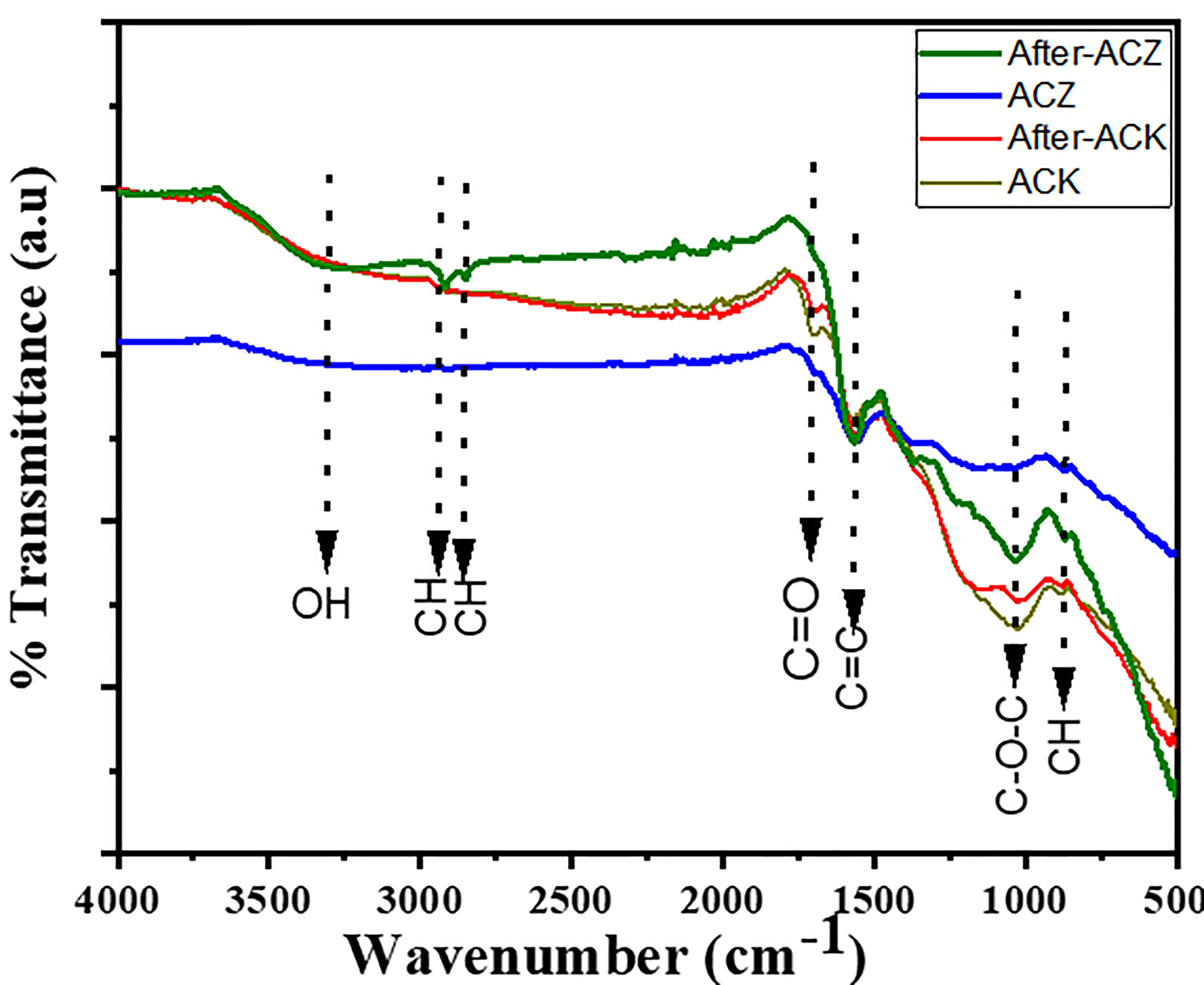


**Fig****. S2.** The FTIR spectroscopy of ACZ, and ACK before and after TC antibiotics adsorption.

**References**

[1] C.P. Okoli, A.E. Ofomaja, Development of sustainable magnetic polyurethane polymer nanocomposite for abatement of tetracycline antibiotics aqueous pollution: Response surface methodology and adsorption dynamics, J. Cleaner Prod. 217 (2019) 42-55.

[2] I.A. Lawal, T.H. Dolla, K. Pruessner, P. Ndungu, Synthesis and characterization of deep eutectic solvent functionalized CNT/ZnCo_2_O_4_ nanostructure: Kinetics, isotherm and regenerative studies on Eosin Y adsorption, J. Environ. Chem. Eng. 7 (2019) 102877.

[3] P.N. Diagboya, F.M. Mtunzi, K.O. Adebowale, R.-A. Düring, B.I. Olu-Owolabi, Comparative empirical evaluation of the aqueous adsorptive sequestration potential of low-cost feldspar-biochar composites for ivermectin, Colloids and Surf. A: Physicochem. Eng. Aspects 634 (2022) 127930.

[4] P.R. Sera, P.N. Diagboya, S.O. Akpotu, F.M. Mtunzi, T.B. Chokwe, Potential of valourized Moringa oleifera seed waste modified with activated carbon for toxic metals decontamination in conventional water treatment, Biores. Technol. Reports 16 (2021) 100881.

[5] T.D. Ntuli, V.E. Pakade, Hexavalent chromium removal by polyacrylic acid-grafted Macadamia nutshell powder through adsorption–reduction mechanism: adsorption isotherms, kinetics and thermodynamics, Chem. Eng. Commun. 207 (2020) 279-294.

[6] N. Boudrahem, S. Delpeux-Ouldriane, L. Khenniche, F. Boudrahem, F. Aissani-Benissad, M. Gineys, Single and mixture adsorption of clofibric acid, tetracycline and paracetamol onto activated carbon developed from cotton cloth residue, Proc. Safety Environ Protec. 111 (2017) 544-559.

[7] X. Sheng, J. Wang, Q. Cui, W. Zhang, X. Zhu, A feasible biochar derived from biogas residue and its application in the efficient adsorption of tetracycline from an aqueous solution, Environ. Res. 207 (2022) 112175.

[8] R. Acosta, V. Fierro, A.M. De Yuso, D. Nabarlatz, A. Celzard, Tetracycline adsorption onto activated carbons produced by KOH activation of tyre pyrolysis char, Chemosphere, 149 (2016) 168-176.

[9] M.J. Ahmed, M.A. Islam, M. Asif, B.H. Hameed, Human hair-derived high surface area porous carbon material for the adsorption isotherm and kinetics of tetracycline antibiotics, Biores. Technol. 243 (2017) 778-784.

[10] M. Islam, B. Hameed, M. Ahmed, W. Khanday, M.A. Khan, F. Marrakchi, Porous carbon–based material from fish scales for the adsorption of tetracycline antibiotics, Biomass Conversion and Biorefinery, (2022) 1-10.

[11] J. Yang, Y. Dou, H. Yang, D. Wang, A novel porous carbon derived from CO_2_ for high-efficient tetracycline adsorption: Behavior and mechanism, Appl. Surf. Sci. 538 (2021) 148110.

[12] M. Wei, F. Marrakchi, C. Yuan, X. Cheng, D. Jiang, F.F. Zafar, Y. Fu, S. Wang, Adsorption modeling, thermodynamics, and DFT simulation of tetracycline onto mesoporous and high-surface-area NaOH-activated macroalgae carbon, J. Hazard. Mat. 425 (2022) 127887.

[13] M.T. Islam, A.H.M.G. Hyder, R. Saenz-Arana, C. Hernandez, T. Guinto, M.A. Ahsan, B. Alvarado-Tenorio, J.C. Noveron, Removal of methylene blue and tetracycline from water using peanut shell derived adsorbent prepared by sulfuric acid reflux, J. Environ. Chem. Eng. 7 (2019) 102816.

[14] M.A. Ahsan, M.T. Islam, C. Hernandez, E. Castro, S.K. Katla, H. Kim, Y. Lin, M.L. Curry, J. Gardea-Torresdey, J.C. Noveron, Biomass conversion of saw dust to a functionalized carbonaceous materials for the removal of Tetracycline, Sulfamethoxazole and Bisphenol A from water, J. Environ. Chem. Eng.6 (2018) 4329-4338.

[15] H. Sayğılı, F. Güzel, Effective removal of tetracycline from aqueous solution using activated carbon prepared from tomato (Lycopersicon esculentum Mill.) industrial processing waste, Ecotoxicol. Environ. Safety 131 (2016) 22-29.

[16] Y. Cai, L. Liu, H. Tian, Z. Yang, X. Luo, Adsorption and desorption performance and mechanism of tetracycline hydrochloride by activated carbon-based adsorbents derived from sugar cane bagasse activated with ZnCl_2_, Molecules, 24 (2019) 4534.

[17] S. Fan, Y. Wang, Y. Li, Z. Wang, Z. Xie, J. Tang, Removal of tetracycline from aqueous solution by biochar derived from rice straw, Environ. Sci. Poll. Res. 25 (2018) 29529-29540.

[18] C. Zhao, J. Ma, Z. Li, H. Xia, H. Liu, Y. Yang, Highly enhanced adsorption performance of tetracycline antibiotics on KOH-activated biochar derived from reed plants, RSC Adv. 10 (2020) 5066-5076.
